# Chelating drug-induced labile Zn^2+^ with nanoparticle-encapsulated TPEN at low dose enhances lung cancer chemotherapy through inhibiting ABCB1

**DOI:** 10.1016/j.isci.2024.111072

**Published:** 2024-10-05

**Authors:** Linlin Wang, Chen Ni, Kaili Zhang, Yuanyuan Yang, Ruoyang Chen, Xiaohan Lou, Yan Yan, Kexin Li, Ya Dong, Xiaohan Yao, Jiajia Wan, Xixi Duan, Fazhan Wang, YongJuan Li, Zhihai Qin

**Affiliations:** 1 Medical Research Center, Henan China–Germany International Joint Laboratory of Tumor Immune Microenvironment and Disease, the First Affiliated Hospital of Zhengzhou University, Zhengzhou University, Zhengzhou, Henan 450052, China; 2 The Center of Infection and Immunity, Academy of Medical Sciences, Zhengzhou University, Zhengzhou, Henan 450001, China

**Keywords:** Biological sciences, Biomaterials, Cancer, Drug delivery system

## Abstract

Chemotherapy resistance is still a great challenge for clinical treatment of lung cancer. Here, we found that doxorubicin (DOX) induced an increase of labile Zn^2+^ in lung cancer cells, and these labile Zn^2+^ protected tumor cells against DOX cytotoxicity. Nanoparticles encapsulating N,N,N′,N′-Tetrakis (2-pyridylmethyl)-ethylenediamine (TPEN) were then constructed to chelate labile Zn^2+^ for tumor therapy. Application of nanoparticle-encapsulated TPEN at low dose not only avoided severe side effects caused by removing physiological Zn^2+^ but also effectively chelated drug-induced labile Zn^2+^, and thereby enhanced DOX cytotoxicity. Mechanistically, nanosized TPEN inhibits ABCB1-mediated drug export potentiated by drug-induced labile Zn^2+^. Finally, the results unraveled that nanosized TPEN at low dose endowed DOX with the killing ability on resistant tumor cells. Taken together, our results demonstrate that chelating drug-induced labile Zn^2+^ by nanosized TPEN at low dose enhances lung cancer chemotherapy by inhibiting ABCB1, providing a feasible strategy to overcome chemoresistance in lung cancer.

## Highlights

• Labile zinc induced by DOX protects lung cancer cells against DOX cytotoxicity
• Labile zinc promotes drug export by regulating ABCB1 activity in lung cancer cells
• Nanosized TPEN at low dose chelates labile zinc and promotes DOX cytotoxicity
• Nanosized TPEN plus DOX effectively suppresses the growth of resistant lung cancer cells

## Graphical abstract

**Figure:**
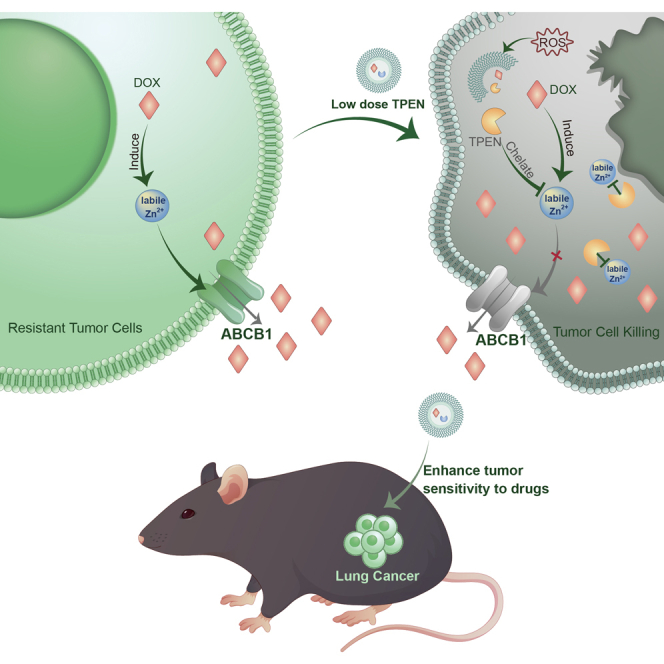

## Introduction

Lung cancer currently leads to the highest number of deaths among all patients with cancer, with the incidence and mortality ranking first for men and in the top three for women.^1^ Chemotherapy, surgery, radiotherapy, and immunotherapy are primary treatments for lung cancer. Chemotherapy stands as a primary option for patients grappling with advanced and metastatic tumors, especially when surgical interventions are no longer viable.^2^^,^^3^ Regrettably, chemotherapy resistance frequently emerges among lung cancer patients, presenting a formidable challenge. Chemotherapy resistance is generally caused by changes in tumor epigenetics, drug metabolism, immune escape, and related signaling pathways.^4^^,^^5^ During the course of tumor progression and treatment, fluctuations and dyshomeostasis in metal ions including Zn^2+^, have been documented.^6^ Nevertheless, how zinc dysregulation contributes to chemotherapy resistance and the corresponding translational potential remain largely undiscovered.

Zinc is a vital trace element within animal body, playing multiply physiological roles in DNA synthesis, cell proliferation, and apoptosis.^7^ Zinc homeostasis is tightly regulated by various proteins including two major zinc transporter families, ZIP (also known as SLC39A, 1–14) and ZnT (also known as SLC30A, 1–10) family proteins in human.^8^ Cellular zinc presents as “bound Zn^2+^” that is sequestered within proteins, or “labile Zn^2+^” that is loosely bound by peptides or ligands.^9^ Under stress conditions, such as oxidative stress, there is a remarkable increase of labile Zn^2+^ within cells.^9^^,^^10^ For example, hydrogen peroxide (H_2_O_2_) treatment elevates cellular labile Zn^2+^.^9^ Chemotherapeutic drugs such as doxorubicin (DOX) often induce reactive oxygen species (ROS) in tumor cells.^11^^,^^12^ However, whether chemotherapeutic drugs affect zinc homeostasis lacks studies.

Zinc has long been thought to be a target for tumor therapy, but specific strategies and underly mechanisms are still in controversy. Zinc complement have been explored to alleviate therapy-related side effects, and to restrict tumor growth.^13^^,^^14^ In contrast, chelating zinc using chelators such as TPEN (N,N,N′,N′-Tetrakis (2-pyridylmethyl)-ethylenediamine), has also been investigated for tumor treatment. TPEN has high affinity with Zn^2+^ and is able to chelate labile Zn^2+^, therefore being used to induce tumor cell death,^15^^,^^16^ and likewise, TPEN may also cause severe damages to normal cells.^17^^,^^18^ This complexity is possibly untangled along with the understanding of specific roles of Zn^2+^ in tumor progression and therapy. We have noted that labile Zn^2+^ may play a role in chemotherapy resistance.^19^ Taylor et al. reported that ZIP7 upregulation is associated with tamoxifen resistance in breast cancer.^20^ Therefore, we suspected that chelating labile Zn^2+^ might be a feasible way to counteract chemotherapy resistance.

In this study, we find that labile Zn^2+^ in lung cancer cells could be induced by chemotherapeutic drug treatment, and chelating labile Zn^2+^ by nanosized TPEN at low dose effectively promoted the killing of cancer cells by chemotherapy. Instead of intending to directly kill tumor cells by single TPEN, we adopted a relatively low dose of TPEN that is adequate to chelating drug-induced labile Zn^2+^ in tumor cells, and is less toxicity on normal cells, to minimize potential side effects that caused by removing physiological Zn^2+^. Considering the poor solubility of TPEN, we designed and constructed nanoparticles to encapsulate TPEN (NP@TPEN). As anticipated, nanoparticle-encapsulated TPEN at low dose effectively chelated drug-induced labile Zn^2+^ in tumor cells, and enhanced the cytotoxicity of DOX on tumor cells. The underlying molecular mechanism and whether nanosized TPEN at low dose could help DOX to kill insensitive tumor cells were further investigated. Our results demonstrate that chelating drug-induced labile Zn^2+^ by nanoparticle-encapsulated TPEN at low dose enhances lung cancer chemotherapy, having important translational potential to overcome chemoresistance in lung cancer therapy.

## Results

### Drug-induced labile Zn^2+^ is protective for lung cancer cells with chemotherapy

**Figure 1:**
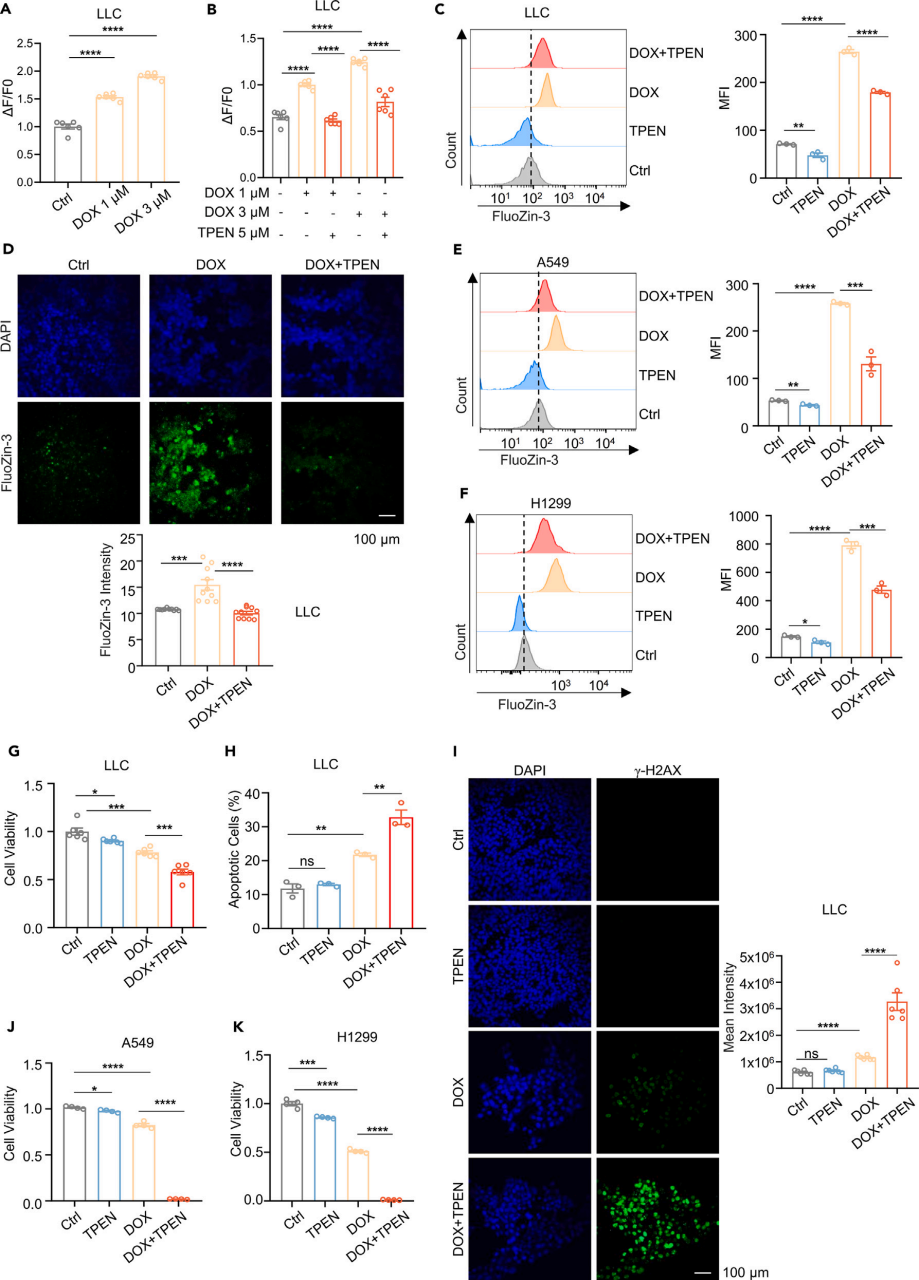
Doxorubicin (DOX) induced the increase of labile Zn^2+^ that was protective against the cytotoxicity of DOX in tumor cells (A) The levels of active zinc were labeled with FluoZin-3 staining (*n* = 6). (B) After adding DOX and TPEN for 24 h, and zinc levels were detected with FluoZin-3 stained (*n* = 6). (C, E, and F) Treatment with 5 μM TPEN, 1 μM DOX, and DOX@TPEN (1 μM DOX combined with 5 μM TPEN) for 24 h, intracellular zinc content was assessed by FluoZin-3 staining and flow cytometry (*n* = 3). (D) Confocal laser scanning microscopy (CLSM) images depicting labile zinc expression by FluoZin-3 staining after treatment with 1 μM DOX and 5 μM TPEN for 24 h (*n* = 10 mice per group). (G, J, and K) Cell viability assessed after treatment with 5 μM TPEN and 1 μM DOX for 24 h (G: *n* = 6; J, K: *n* = 4). (H) Cell apoptosis by flow cytometric analyses treatmented with 5 μM TPEN and 1 μM DOX for 24 h (*n* = 3). (I) CLSM images of γ-H2AX expression after treatment with 5 μM TPEN and 1 μM DOX for 24 h (*n* = 6 pictures per group). Data represents the mean ± SEM of at least three replicates per condition. t test was employed for statistical analysis. Scale bar is 100 μM ∗*p* < 0.05; ∗∗*p* < 0.01; ∗∗∗*p* < 0.001; ∗∗∗∗*p* < 0.0001; ns, not significant.

To examine the effect of chemotherapeutic drugs on zinc homeostasis in lung cancer, we treated tumor cell lines with different doses of drugs in zinc-free culture medium. The level of intracellular labile Zn^2+^ was determined using FluoZin-3 staining. The results showed that labile Zn^2+^ in LLC cells increased after treatment with DOX and paclitaxel (PTX) compared with those in cells without treatment (Figures 1A and S1A). Similar results were obtained for human lung cancer cell lines A549 and H1299 (Figures S1B and S1C). We also used TPEN to reverse drug-induced increase of labile Zn^2+^. The results showed that TPEN (5 μM) effectively reduced DOX-induced labile Zn^2+^ in LLC, A549, and H1299 cells (Figures 1B–1F). These results demonstrate that chemotherapeutic drugs induce the increase of intracellular labile Zn^2+^.

An interesting question is whether drug-induced labile Zn^2+^ is cytotoxic or protective for tumor cells.

We noted that addition of TPEN (5 μM) reduced DOX-induced labile Zn^2+^ that still higher than those in cells without treatment (Figures 1C, 1E, and 1F). Thus, the lung cancer cells were treated with this condition, and cell proliferation, apoptosis, and DNA damage were examined. The results showed that both TPEN (5 μM) and DOX (1 μM) inhibited the cell viability of LLC (Lewis lung carcinomaer) cells examined by CCK8 assay (9.73 ± 0.01% for TPEN, 21.72 ± 0.02% for DOX), while the combination of TPEN and DOX increased the inhibition of cell viability of LLC (42.05 ± 0.03%) than each single drug (Figure 1G). We also measured cell proliferation using Ki-67 staining, and the results showed that both TPEN and DOX inhibited Ki-67 expression in LLC cells (10.27 ± 2.15% for TPEN, 37.95 ± 1.38% for DOX), while the combination of TPEN and DOX enhanced the inhibition of Ki-67 expression (73.65 ± 1.58%) than each single drug (Figure S1D). Interestingly, TPEN alone did not induced cell apoptosis evaluated with Annexin-V staining, or DNA damage evaluated with γ-H2AX expression (Figures 1H and 1I), but TPEN dramatically enhanced DOX-induced cell apoptosis and DNA damage in LLC cells (42.12 ± 0.51% for DOX, 88.81 ± 2.14% for DOX+TPEN) (Figure 1H), suggesting that TPEN enhanced the killing of tumor cells by DOX. In addition, we treated two human lung cancer cell lines, A549 and H1299, for validation, and similar results were observed (Figures 1J, 1K, S1E, and S1F). These results indicate that drug-induced labile Zn^2+^ is protective for tumor cells.

### Construction of nanoparticle-encapsulated TPEN

**Figure 2:**
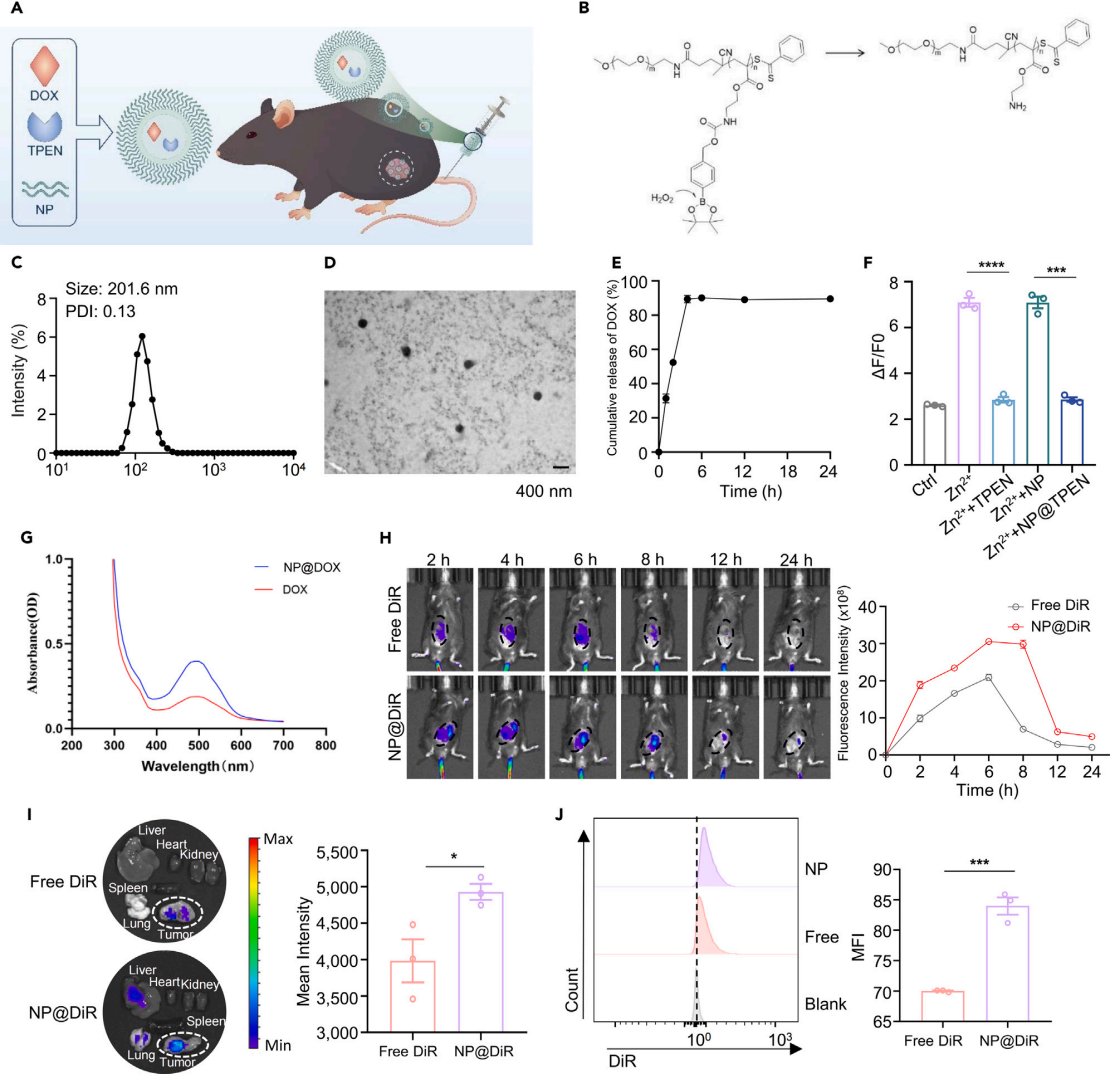
Construction of nanoparticles encapsulating TPEN and DOX (A) *In vivo* therapeutic mode diagram of nanomedicine. (B) ROS responsive mechanism of nanomaterials. (C) Determination of nanoparticle size using dynamic light scattering (DLS). (D) Transmission electron microscopy (TEM) image depicting nanoparticle morphology (Scale bar, 400 nM). (E) Cumulative release of DOX (*n* = 3). (F) Verified the successful loading of TPEN onto nanoparticles. The concentration of the zinc, TPEN, NP@TPEN, and nanoparticle shell (NP) was 5 μM. In addition, all groups were treated with 5 mM H_2_O_2_ for 24 h (releasing TPEN encapsulated in the nanoparticles), and then stained with FluoZin-3 and detected (*n* = 3). (G) UV-vis spectra of DOX and NP@DOX. (H) Administration of free DiR and NP@DiR through the tail vein in tumor-bearing mice, with observation of DIR accumulation in tumor tissue at different time points. (I) Examination of the residual presence of DiR in various organs and tumor tissues. (J) Flow cytometry employed to detect the residual level of DiR in tumor tissue after 24 h. Data represents the mean ± SEM of at least three replicates per condition. (H–J): *n* = 3–6 mice per group. t test was employed for statistical analysis. ∗*p* < 0.05; ∗∗*p* < 0.01; ∗∗∗*p* < 0.001; ∗∗∗∗*p* < 0.0001; ns, not significant.

Because chelating drug-induced labile Zn^2+^ by TPEN enhanced the killing of tumor cells by chemotherapeutic drug, we attempted to deliver TPEN to enhance tumor chemotherapy. For this regard, we designed and constructed ROS-responsive nanoparticles to encapsulate TPEN (NP@TPEN), DOX (NP@DOX), and the combination of DOX with TPEN (NP@DOX@TPEN) for tumor therapy (Figure 2A). When the nanoparticles are engulfed into tumor cells with high ROS, the self-assembly structure of nanoparticles would be destroyed, and the drug be released. The minimum ROS concentration that can achieve response is 100 μM (Figure 2B). The size of constructed nanoparticles is around 201.6 nm (Figures 2C and 2D). Through analyzing by spectrofluorimetry, the drug loading of DOX (λ_ex_ = 480 nm, λ_em_ = 590 nm) is 9% (Figure 2E). By high-performance liquid chromatography (HPLC) analysis, the drug loading capacity of TPEN is 12%. We added NP@TPEN to Zn^2+^ solution, and the results showed that Zn^2+^ signals were inhibited suggesting the successful loading of TPEN into nanoparticles (Figure 2F). The spectral absorption of NP@DOX was analyzed, and the results showed that there was an apparent absorption at 560 nm indicating the successful loading of DOX into nanoparticles (Figure 2G). Next, we injected nanoparticles-encapsulated a fluorescent dye DiR (NP@DiR) or free DiR (1,1′-Dioctadecyl-3,3,3′,3′-tetraMethylindotricarbocyanine iodide) into the tail veins of tumor-bearing C57BL/6 mice. Live imaging of animals was performed at different time post injection. The results showed that the fluorescence was mostly accumulated in the tumor sites, and the fluorescence in tumor was higher in mice with NP@DiR injection than that with free DiR injection (Figure 2H). After 24 h of filming, the heart, liver, spleen, lungs, kidneys, and tumor tissues of the mice were collected for photography to observe the residual DiR status in various organs. The results showed that nanoparticles effectively delivered DiR to the tumor sites (Figure 2I). Subsequently, the tumor tissues were digested into a single-cell suspension, and the fluorescence in tumor cells was evaluated by flow cytometry. The results showed that nanoparticles delivered DiR into the tumor cells (Figure 2J).

### Nanosized TPEN at low dose enhances the cytotoxicity of DOX

**Figure 3:**
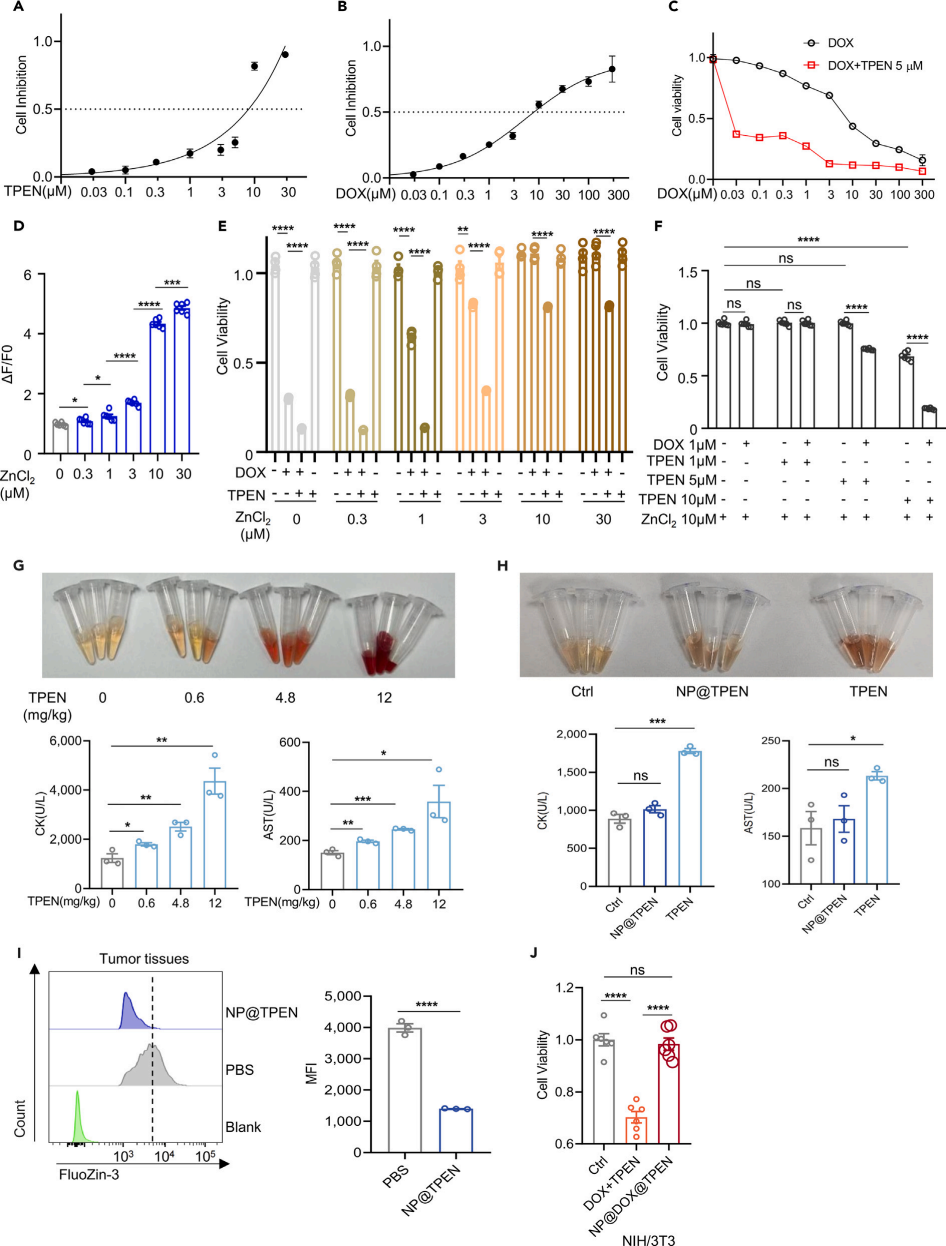
TPEN at low dose enhanced the cytotoxicity of DOX and avoided severe side effects (A–C) The evaluation of LLC cells viability after 24 h of treatment with different concentrations of TPEN, DOX, and the combined treatment of DOX with 5 μM TPEN (*n* = 4–5). (D) Firstly, LLC cells were starved for 2 h, then different concentrations of ZnCl_2_ were added for 1 h, and then the unabsorbed zinc was removed. The cells were washed with PBS and then stained with FluoZin-3 (*n* = 6). (E) After the cells absorbed different concentrations of zinc, the killing effects of DOX and TPEN on them were detected (*n* = 4). (F) After the cells were treated with 10 μM ZnCl_2_, 1 μM DOX, and different concentrations of TPEN were added to observe cell viability (*n* = 6). (G) Assessment of the impact of various TPEN concentration on serum indicators in mice. (H) The effect of TPEN and NP@TPEN on serum indicators in mice. (I) Detected the zinc level in tumor tissue after injecting NP@TPEN. (J) The inhibition effects of drugs and nanoparticles loaded drugs were compared after adding in NIH/3T3 cells for 24 h (*n* = 6). Data represents the mean ± SEM of three replicates/condition. (G–I): *n* = 3 mice per group. T test was utilized for statistical analysis. ∗*p* < 0.05; ∗∗*p* < 0.01; ∗∗∗*p* < 0.001; ∗∗∗∗*p* < 0.0001; ns, not significant.

Because Zn^2+^ has important physiological functions in normal cells, chelation of Zn^2+^ by TPEN may be toxic to normal cells, such as neurons.^21^ Our aforementioned results showed that TPEN at 5 μM did not induce cell apoptosis and DNA damage but enhanced the toxicity of DOX on tumor cells, indicating that it is possible to use TPEN to enhance chemotherapy instead of directly inducing tumor cell death. Thus, we intended to use TPEN at low dose to avoid the side effects as much as possible. Firstly, we observed the inhibiting effects of different doses of TPEN on LLC cells. As shown in Figure 3A, the inhibitory effect of TPEN on LLC cells gradually enhanced along with the increase of concentrations, and the half maximal inhibitory concentration was 8.412 μM. Therefore, we adopted TPEN at 5 μM to combine with different doses of DOX. The results showed that TPEN enhanced the inhibitory effect of DOX even at a very low dose (0.03 μM) (Figures 3B and 3C). According to the aforementioned results, the change of intracellular labile zinc produced by tumor cells upon drug stimulation is crucial for tumor chemotherapy resistance. Therefore, we incubated tumor cells with different concentrations of ZnCl_2_ to generate tumor cells with various levels of labile zinc (Figure 3D). Then drugs were added onto these tumor cells. The results showed that the more labile zinc absorbed by cells, the more resistant to DOX, and the addition of TPEN could enhance the killing effect of DOX even on the resistant tumor cells (Figure 3E). Subsequently, we further optimized TPEN doses in resistant tumor cells with 10 μm zinc treatment. The results showed that 1 μm DOX did not inhibit the growth of tumor cells. When TPEN was below 5 μm, TPEN had no killing effect, but DOX combined with TPEN at 5 μm inhibited the growth of tumor cells. When TPEN reached 10 μm, itself caused damage to tumor cells (Figure 3F).

Considering the dosage of TPEN for *in vivo* treatment in mice, we roughly estimated that TPEN at 5 μM *in vitro* was approximately equal to 0.52 mg/kg in mice. To examine the potential toxicity of TPEN *in vivo*, we injected different doses of TPEN (0, 0.6, 4.8, 12 mg/kg) into C57BL/6 mice through the tail vein and the serum was collected 12 h later. The results showed that higher doses of TPEN (4.8, 12 mg/kg) significantly induced hemolysis, heart and liver damages. In contrast, low dose of TPEN (0.6 mg/kg) did not produce severe hemolysis, nor did it cause severe damage to the myocardium (creatine kinase, CK, ref. 0–2070.55 U/L) and liver function (aspartate transaminase, AST, ref. 36.31–235.48 U/L) of mice, although the level of CK and AST significantly increased when compared to the untreated mice (Figure 3G). Then, we administered NP@TPEN equal to 0.6 mg/kg TPEN in C57BL/6 mice. The results showed that NP@TPEN induced neither hemolysis, nor myocardial damage, or impaired liver function (Figure 3H), which is similar to that in untreated mice and lower than that in mice with free TPEN. This concentration of TPEN is relatively low compared to the TPEN concentration (1–10 mg/kg) used in other studies to induce tumor cell death.^22^^,^^23^ To confirm the chelation of Zn^2+^ by NP@TPEN (0.6 mg/kg) in tumor, we injected NP@TPEN into LLC tumor-bearing mice. The level of labile Zn^2+^ in tumor cells was examined by flow cytometry 12 h later. The results showed that labile Zn^2+^ in tumor cells with NP@TPEN treatment reduced compared to that with control treatment (Figure 3I). Therefore, in following studies, we used NP@TPEN equal to 0.6 mg/kg TPEN *in vivo* and equal to 5 μM TPEN *in vitro* to combine with chemotherapy. Finally, we conducted experiments in NIH/3T3 cells and the results showed that the nanomaterials did not release drugs to damage cells in normal fibroblasts, implying that the material might has no severe side effects on normal cells (Figure 3J).

**Figure 4:**
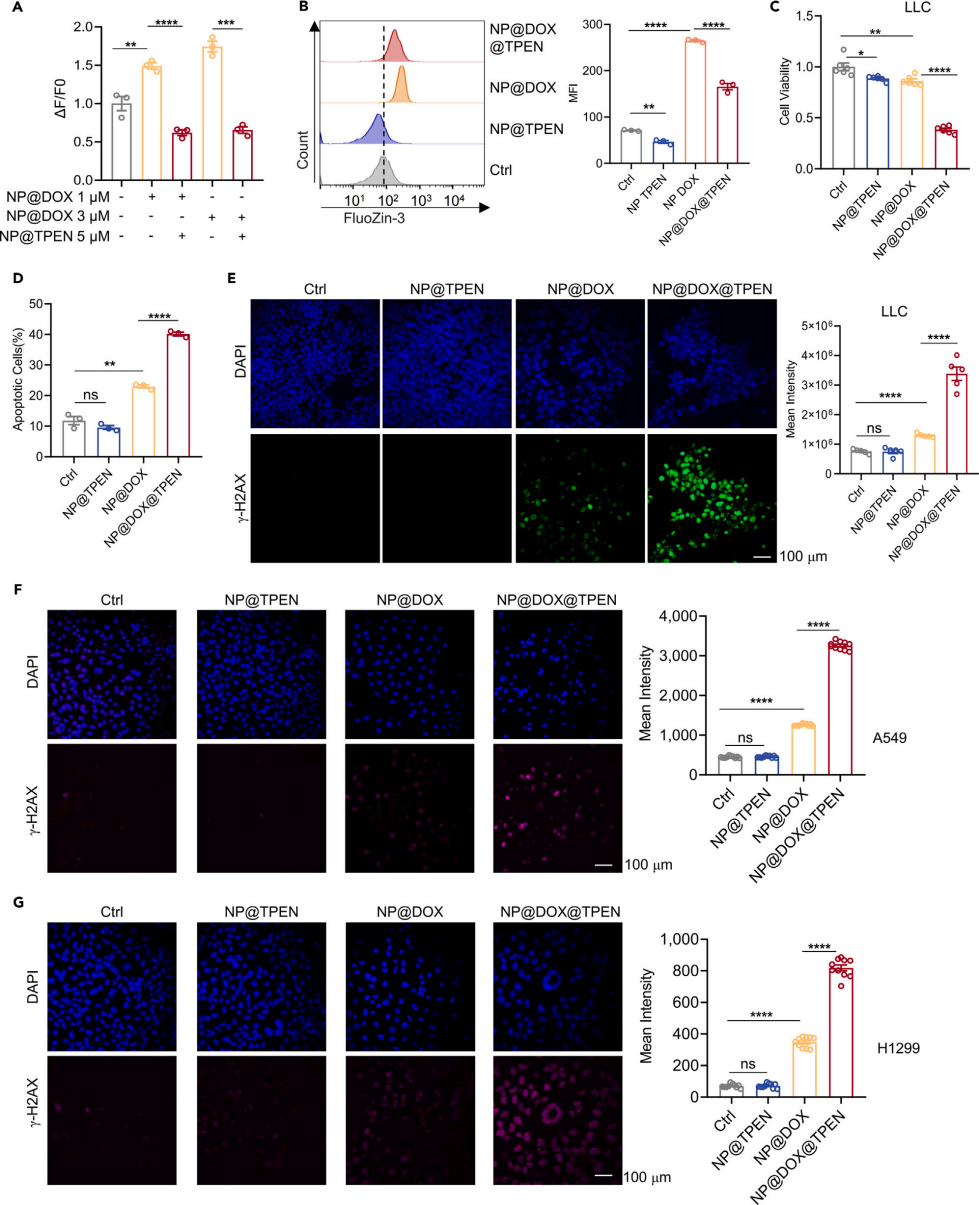
NP@TPEN at low dose reduced drug-induced labile Zn^2+^ and enhanced the cytotoxicity of NP@DOX in lung cancer cells (A) The zinc inside the LLC cells with FluoZin-3 stained were detected (*n* = 3). (B) Flow cytometry analysis of intracellular zinc content in LLC cells treated with 5 μM NP@TPEN, 1 μM NP@DOX, and NP@DOX@TPEN (1 μM NP@DOX combined with 5 μM NP@TPEN) to LLC cells for 24 h, following FluoZin-3 staining (*n* = 3). (C) Cell viability assessed by CCK-8 assay after treatment for 24 h (*n* = 6). (D) Flow cytometric analysis of cell apoptosis in LLC cells (*n* = 3). (E–G) CLSM images of γ-H2AX expression in LLC, A549, and H1299 cells. Data are presented as the mean ± SEM from a minimum of three replicates per condition. (E): *n* = 5 pictures per group; (F and G): *n* = 10 pictures per group. t test was utilized for statistical analysis. Scale bar is 100 μM ∗*p* < 0.05; ∗∗*p* < 0.01; ∗∗∗*p* < 0.001; ∗∗∗∗*p* < 0.0001; ns, not significant.

Next, we examined whether nanosized TPEN retained its ability to chelate labile Zn^2+^ and the enhancement of the cytotoxicity of DOX. The results showed that NP@DOX induced an increase of labile Zn^2+^, which was effectively reduced by NP@TPEN (Figures 4A and 4B). The viability of LLC cells with NP@DOX treatment (86.34 ± 0.02%) was higher than that with NP@DOX@TPEN treatment (38.33 ± 0.01%) (Figure 4C). Similar results were observed for the inhibition of tumor cell proliferation labeled by Ki67 (Figure S2A). More importantly, NP@TPEN alone did not induce cell apoptosis and DNA damage but enhanced NP@DOX-induced cell apoptosis and DNA damage in LLC cells (Figures 4D and 4E). We further confirmed these observation in human lung cancer cells A549 and H1299 (Figures 4F, 4G, and S2B–S2G). These results suggested that nanosized TPEN at low dose enhanced the cytotoxicity of DOX without causing severe side effects.

### Nanosized TPEN inhibits ABCB1-mediated drug export potentiated by drug-induced labile Zn^2+^

**Figure 5:**
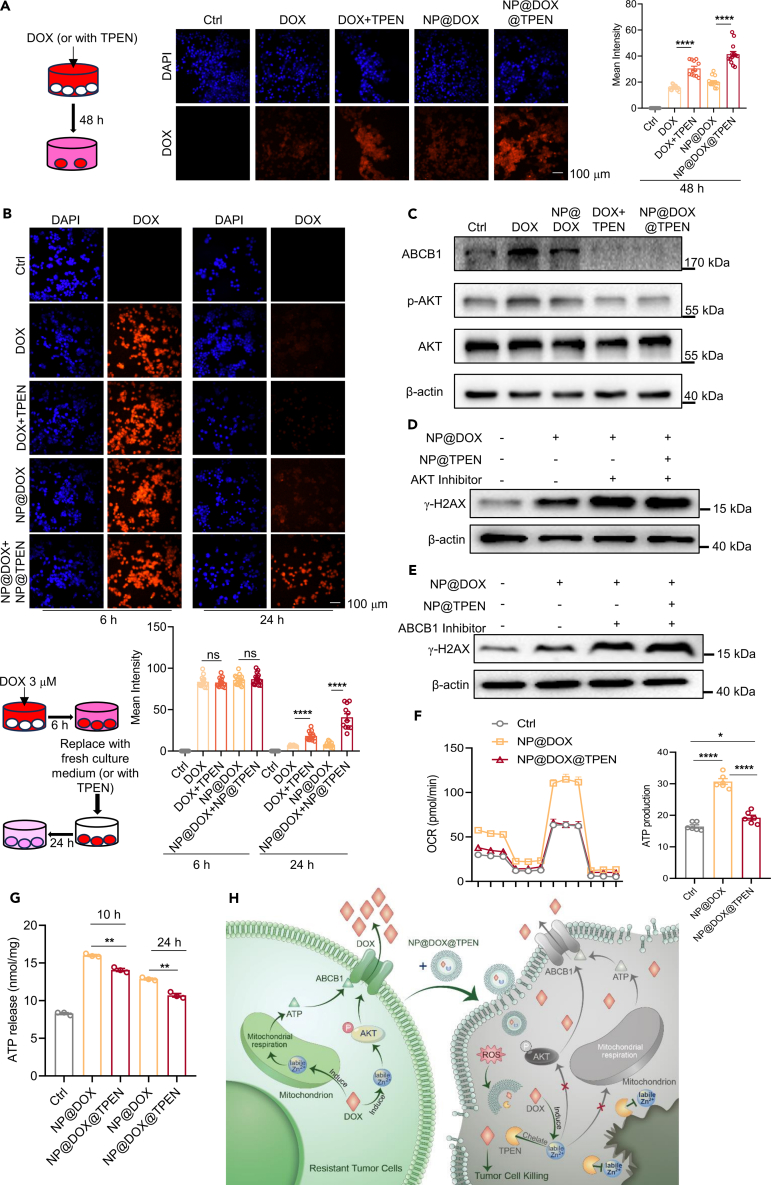
NP@TPEN inhibited ABCB1-mediated drug export potentiated by drug-induced labile Zn^2+^ (A) CLSM images illustrating the intracellular accumulation of DOX in LLC cells (*n* = 16 pictures per group). (B) CLSM images of the residual DOX in LLC cells (*n* = 16 pictures per group). (C) Western blot analysis in LLC cells, after adding 1 μM DOX, 1 μM NP@DOX, DOX@TPEN (1 μM DOX combined with 5 μM TPEN), and NP@DOX@TPEN (1 μM NP@DOX combined with 5 μM NP@TPEN) for 24 h. (D and E) Western blot analysis of γ-H2AX expression after treatment with 1 μM NP@DOX, NP@DOX@TPEN (1 μM NP@DOX combined with 5 μM NP@TPEN), NP@DOX@TPEN with 25 μM AKT inhibitor, and NP@DOX@TPEN with 0.1 μM ABCB1 inhibitor in LLC cells. (F) Seahorse experiment measuring mitochondrial respiration and ATP production for 24 h in LLC cells (1 μM NP@DOX, 5 μM NP@TPEN) (*n* = 6). (G) After adding NP@DOX (1 μM), NP@DOX@TPEN (1 μM NP@DOX combined with 5 μM NP@TPEN) to LLC cells, ATP release was detected at different time points (*n* = 3). (H) The increased labile zinc induced by DOX enhances the functions of ABCB1 through two ways to resist drug toxicity, while TPEN can reverse this process. Data are presented as the mean ± SEM from a minimum of three replicates per condition. t test was utilized for statistical analysis. Scale bar is 100 μM ∗*p* < 0.05; ∗∗*p* < 0.01; ∗∗∗*p* < 0.001; ∗∗∗∗*p* < 0.0001; ns, not significant.

Next, we explored the mechanism underlying the enhancement of DOX cytotoxicity by NP@TPEN in LLC cells. In a previous study, we reported that Zn^2+^ stimulated ABCB1-mediated drug export.^19^ Drug-induced labile Zn^2+^ might also potentiate ABCB1-mediated drug export in tumor cells. Therefore, we measured the accumulation of DOX in tumor cells with different treatments using a confocal microscopy. The results showed that DOX accumulated in LLC cells over 24 h when treated with NP@DOX. In combination with NP@TPEN, tumor cells exhibited increased DOX accumulation (Figure 5A). Subsequently, drug excretion tests were conducted. NP@DOX was added onto LLC cells for 6 h and then removed by changing fresh culture medium. NP@TPEN was added at this time and at 24 h, remaining DOX fluorescence was measured. The results showed that DOX retained in the tumor cells was almost totally expelled out of the cells (8.91 ± 0.69% left) with NP@DOX treatment, but the cells with NP@TPEN treatment still kept large amount of DOX (46.97 ± 4.40% left) (Figure 5B). Moreover, we found that NP@DOX treatment increased ABCB1 expression and activated its upstream AKT, which was inhibited by NP@TPEN (Figure 5C). When the inhibitors of AKT or ABCB1 were included, NP@TPEN could not enhance NP@DOX-induced DNA damage indicated by γ-H2AX staining (Figures 5D, 5E, S3A, and S3B). These results suggested that the enhancement of cytotoxicity of NP@DOX by NP@TPEN was dependent on the inhibition of ABCB1-mediated drug export.

ABCB1 requires ATP to pump drugs out of the cells.^24^ We further examined whether NP@TPEN affected ATP production in tumor cells with chemotherapy. Using a Seahorse metabolic analyzer, tumor cell mitochondria respiration was detected. Upon NP@DOX treatment, LLC cells enhanced the mitochondrial respiration to increase ATP production, which was suppressed by NP@TPEN (Figure 5F). In addition, ATP release from tumor cells with different treatment was also examined. The results showed that NP@TPEN suppressed the increase of ATP production in LLC cells with NP@DOX treatment (Figure 5G). These results suggested that the suppression of ATP production by NP@TPEN might also contribute to the inhibition of ABCB1-mediated drug export in tumor cells with chemotherapy (Figure 5H).

### Nanosized TPEN at low dose enhances lung cancer chemotherapy *in vivo*

**Figure 6:**
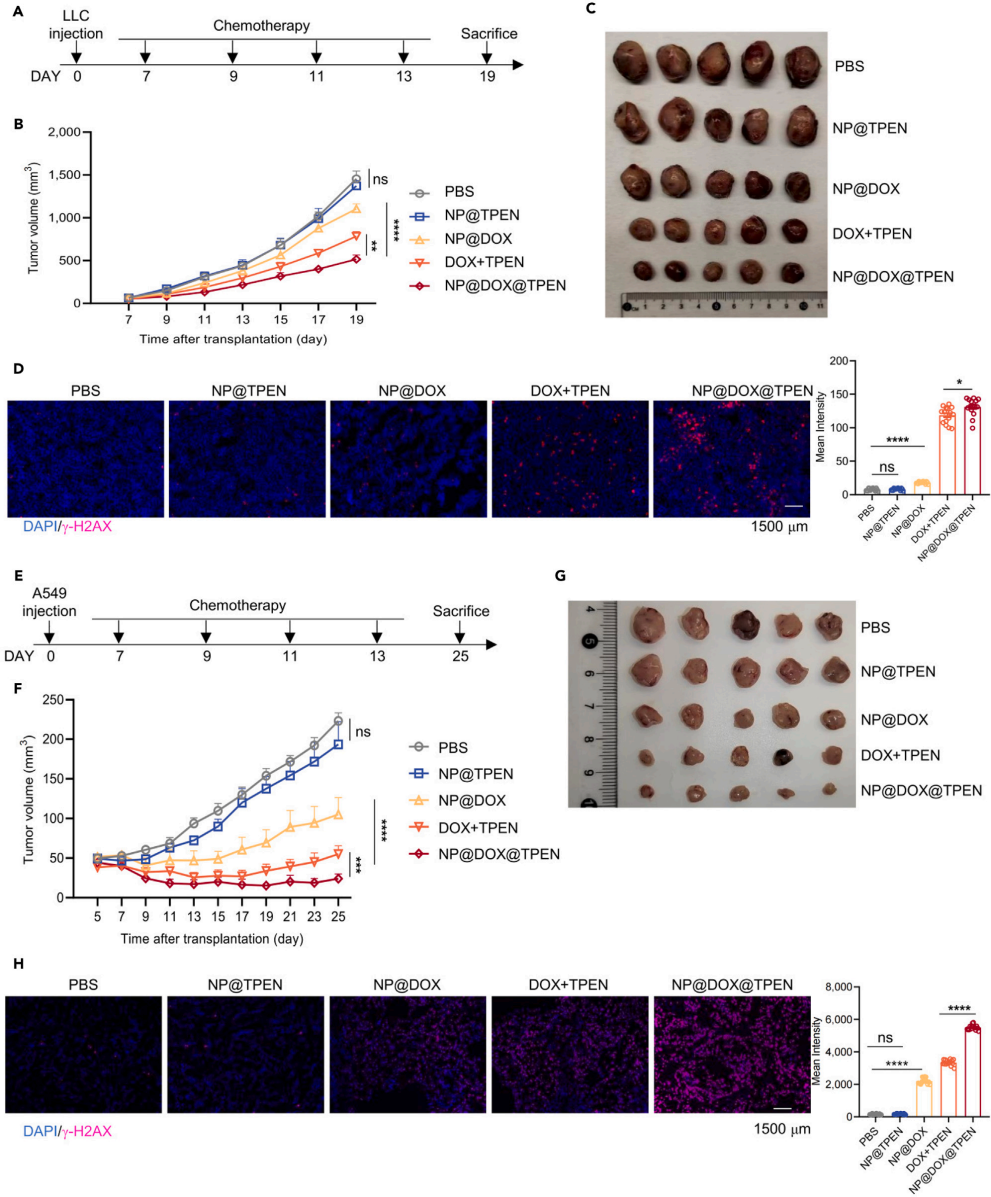
NP@TPEN enhanced chemotherapy of lung cancer *in vivo* (A) Time schedule outlining the treatments administered to C57BL/6 mice. (B and C) Tumor volume measurements over the 19-day observation period. (D) Immunofluorescence images of tumor sections stained with γ-H2AX (Scale bar, 1,500 μM). (E) Time schedule of treatments in nude mice. (F and G) Tumor volume recorded over the 25-day observation period. (H) Immunofluorescence images of tumor sections stained with γ-H2AX (Scale bar, 1,500 μM). The data represent the mean ± SEM from at least three replicates per condition. (B, C, F, and G): *n* = 5 mice per group; (D and H): *n* = 15 pictures per group. (B) and (F) was analyzed by two way ANOVA tests, and (D) and (H) were analyzed by t tests. ∗*p* < 0.05; ∗∗*p* < 0.01; ∗∗∗*p* < 0.001; ∗∗∗∗*p* < 0.0001; ns, not significant.

To explore the therapeutic effect of the nanomedicine *in vivo*, we transplanted LLC cells to C57BL/6 mice. The mice were treated with indicated treatments seven day after transplantation, followed by treatment every other day for a total of four times (DOX and nanoparticle-encapsulated DOX: 5 mg/kg; TPEN and nanoparticle-encapsulated TPEN: 0.6 mg/kg) (Figure 6A). The results showed that NP@TPEN did not affect tumor growth, but NP@TPEN enhanced the inhibition of tumor growth by NP@DOX (Figures 6B and 6C). DNA damage indicated by γ-H2AX staining in tumor sections was not significantly affected by NP@TPEN, but NP@TPEN enhanced NP@DOX-induced DNA damage (Figure 6D). In addition, the nanomedicine treatment did not induce apparent damages to the organs of mice including the heart, liver, spleen, lungs, and kidney, compared to the control group (Figure S4A), suggesting that it was relatively safe to apply NP@TPEN in tumor chemotherapy.

To explore the application of NP@TPEN with chemotherapy in human lung cancer cells, A549 cells were subcutaneously transplanted into nude mice. The mice received treatment once every other day for a total of four times as aforementioned. In consistence, NP@TPEN enhanced the inhibition of tumor growth of A549 by NP@DOX, and promoted DNA damage in tumor induced by NP@DOX (Figures 6E–6H). These results suggested that nanosized TPEN at low dose effectively enhanced lung cancer chemotherapy *in vivo*.

### Nanosized TPEN at low dose endows DOX with the killing ability on tumor cells that are insensitive to chemotherapy

**Figure 7:**
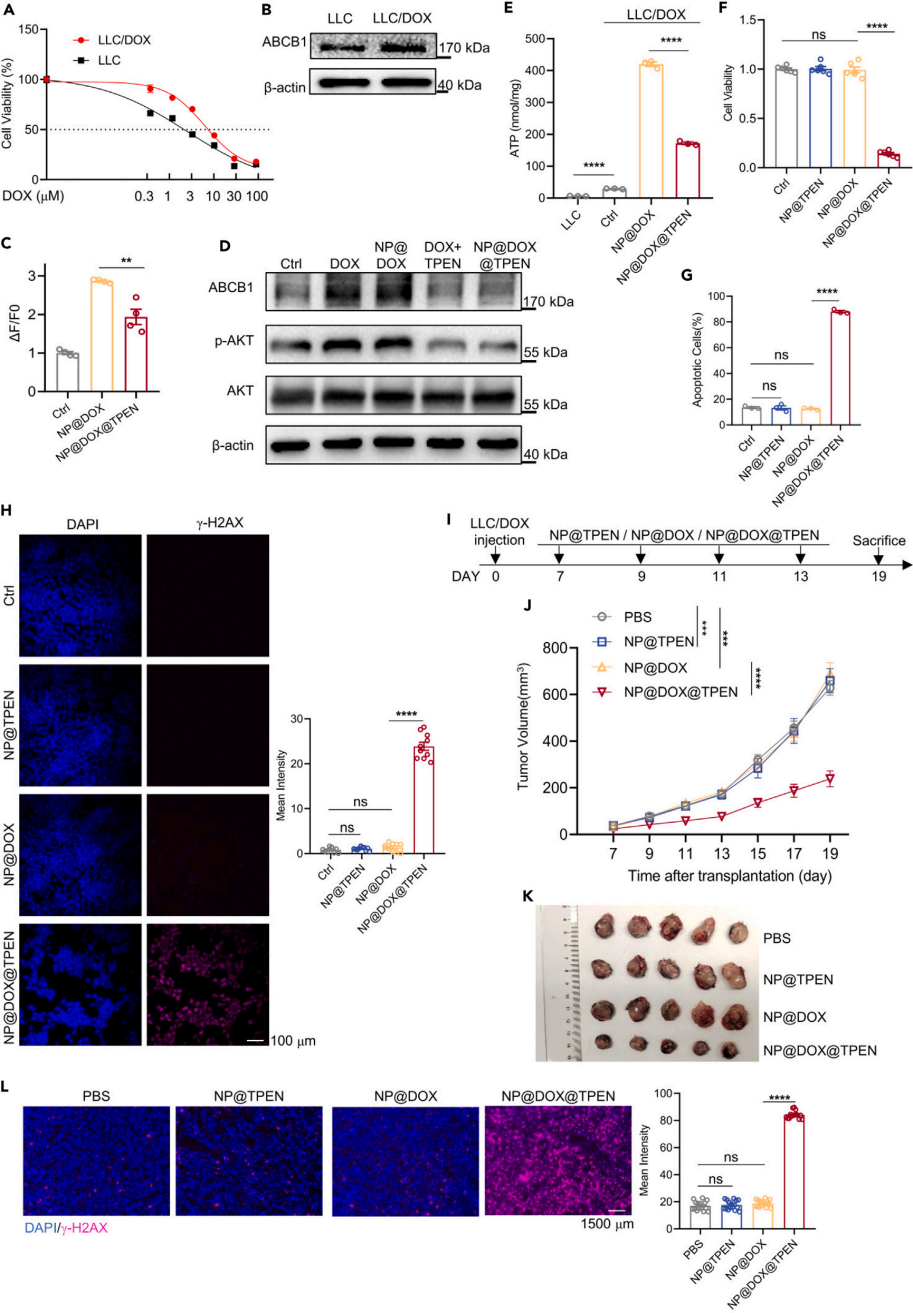
NP@TPEN sensitized resistant LLC/DOX cells to chemotherapy (A) The sensitivity of LLC and LLC/DOX cells to different concentrations of DOX for 24 h. (B) Western blot analysis of ABCB1 expression. (C) The FluoZin-3 staining in LLC/DOX cells to detect intracellular zinc levels (*n* = 4). (D) Western blot analysis of ABCB1, p-AKT, and AKT expression. (E) Measurement of ATP levels in LLC cells and LLC/DOX cells for 24 h. (F) CCK-8 for detecting cell viability (*n* = 6). (G) Cell apoptosis by flow cytometric analyses in LLC/DOX cells for 24 h. (H) CLSM images of γ-H2AX expression LLC/DOX cells (*n* = 10 pictures per group, Scale bar is 100 μM). (I) Time schedule outlining the treatments administered to C57BL/6 mice. (J and K) Tumor volume within the 19-day observation period (*n* = 5 mice per group, Scale bar is 1,500 μM). (L) Immunofluorescence images of tumor sections stained with γ-H2AX (*n* = 15 pictures per group). The data represent the mean ± SEM from at least three replicates per condition. (A, E, and G): *n* = 3. (J) was analyzed by two-way ANOVA tests, and others were analyzed by t tests. ∗*p* < 0.05; ∗∗*p* < 0.01; ∗∗∗*p* < 0.001; ∗∗∗∗*p* < 0.0001; ns, not significant.

To investigate whether NP@TPEN could enhance the killing of resistant tumor cells by chemotherapy, LLC cells that were insensitive to DOX were screened. LLC cells were exposed to DOX at low dose (0.3 μM) for 24 h, and then recovered for 2 days. Following four rounds of screening, the resulted cells were designated as LLC/DOX cells. When LLC/DOX cells were treated with 1 μM DOX, it was observed that 1 μM DOX had no inhibitory effect on cell viability of LLC/DOX (Figure 7A). LLC/DOX cells expressed more ABCB1 than that in LLC cells (Figure 7B). Interestingly, upon treatment with 1 μM NP@DOX, labile Zn^2+^ in LLC/DOX cells still exhibited an increase (Figure 7C). In consistence, ABCB1, p-AKT, and ATP production were further increased in LLC/DOX cells with NP@DOX treatment (Figures 7D and 7E). When NP@TPEN was applied, drug-induced labile Zn^2+^, ABCB1, p-AKT, and ATP production were generally inhibited in LLC/DOX cells (Figures 7C–7E). Therefore, it was possible that NP@TPEN might sensitize resistant tumor cells to chemotherapy. The results showed that both NP@TPEN and NP@DOX had no inhibitory effects on the cell viability of LLC/DOX, but the combination NP@DOX@TPEN drastically reduced the cell viability of LLC/DOX (14.11 ± 0.01%) (Figure 7F). Moreover, only the combination NP@DOX@TPEN stimulated large amount of cell apoptosis and DNA damage in LLC/DOX cells (Figures 7G and 7H). Next, we validated the therapeutic effects of nanomedicines in LLC/DOX tumor-bearing C57BL/6 mice (DOX and nanoparticle-encapsulated DOX: 5 mg/kg; TPEN and nanoparticle-encapsulated TPEN: 0.6 mg/kg). The results demonstrated that NP@DOX@TPEN effectively reduced the tumor volume, while each single drug had no inhibitory effect on tumor growth (Figures 7I–7L). These results suggested that NP@TPEN effectively sensitized resistant tumor cells to chemotherapeutic drugs.

To examine the effects of NP@TPEN on human drug-resistant lung cancer cells, A549/DDP cells resistant to DDP (cisplatin) were adopted. The results showed that A549/DDP cells were resistant to DOX too, and expressed higher level of ABCB1 than A549 cells (Figures S5A and S5B). NP@DOX treatment increased the intracellular labile Zn^2+^, ABCB1, and p-AKT expression and ATP production in A549/DDP cells, which could be suppressed by NP@TPEN (Figures S5C–S5E). While each single drug had no effects, the combination NP@DOX@TPEN drastically reduced the cell viability, enhanced cell apoptosis, and induced DNA damage in A549/DDP cells (Figures S5F–S5H). Subsequently, A549/DDP cells were transplanted into NOD-SCID (non-obese diabetic Severe combined immunodeficiency) mice and treated with nanomedicine (DOX and nanoparticle-encapsulated DOX: 5 mg/kg; TPEN and nanoparticle-encapsulated TPEN: 0.6 mg/kg). The results showed that NP@DOX@TPEN effectively inhibited A549/DDP tumor growth while each single drug had not inhibitory effects (Figures S5I–S5L). In summary, nanosized TPEN at low dose endows DOX with the killing ability on lung cancer cells that are insensitive to chemotherapy through inhibiting ABCB1.

## Discussion

The issue of chemotherapy resistance, particularly in lung cancer, remains a significant challenge in clinical tumor treatment.^25^ Our findings reveal that DOX induces the increase of labile Zn^2+^ in lung cancer cells that contributes to tumor cells’ resistance to chemotherapy through potentiating ABCB1-mediated drug export. Even in resistant lung cancer cells, this phenomenon is still present. Application of nanoparticle-encapsulated zinc chelator TPEN at low dose enhanced lung cancer chemotherapy and endows DOX with the killing ability on tumor cells that were insensitive to chemotherapy, while avoiding severe side effects caused by removing physiological Zn^2+^. Our results demonstrate that chelating drug-induced labile Zn^2+^ by nanosized TPEN at low dose enhances lung cancer chemotherapy by inhibiting ABCB1, providing a feasible strategy to overcome chemoresistance in lung cancer.

Using TPEN at low dose to sensitize tumor cells, instead of direct killing tumor cells, is a possible way to enhance lung cancer chemotherapy. In most studies and common clinical practices, combined drugs often both have killing effects on tumor cells. For example, the combination therapy of DOX with cisplatin for liver cancer,^26^ as well as the combination therapy of methotrexate, cisplatin, DOX, and ifosfamide for osteosarcoma,^27^ and so on. In most previous studies, TPEN mainly is used at high doses (usually 50–500 μM for *in vitro*, 1–10 mg/kg for *in vivo*) to induce tumor cell death,^22^^,^^23^^,^^28^ and the side effects of TPEN are often ignored. In this study, we used TPEN at a relative low dose (5 μM *in vitro*, 0.6 mg/kg *in vivo*). Although the *in vitro* dose of TPEN has slight inhibition of cell proliferation, it does not affect cell apoptosis of tumor cells. The *in vivo* dose of TPEN has no inhibitory effects on tumor growth. But when TPEN is combined with DOX, the killing of tumor cells by DOX is successfully enhanced. At the same time, using TPEN at low dose avoids severe side effects caused by chelating physiological Zn^2+^ *in vivo*. More important, TPEN is able to sensitize resistant tumor cells to chemotherapy.

Nanosized TPEN at low dose effectively delivers TPEN to tumor sites and avoids severe side effects. Nanomaterials play a crucial role in improving drug targeting to tumor tissues and mitigating toxic side effects. TPEN has been linked to potential cognitive impairment in mice.^29^ TPEN also has poor water solubility and no selectivity for cells. Apoptosis induction is a common mechanism in cancer cells for TPEN, but it may also lead to toxicity in healthy cells.^16^ Nanotechnology-based nanocarriers exhibit higher drug entrapment and delivery efficiency, reduced cytotoxicity, enhanced stability, and improved half-life compared to conventional therapy.^30^ Consequently, various nano formulations have been used in preclinical and clinical settings for various cancers, including breast and liver cancers.^31^^,^^32^ Leveraging these characteristics, the nanomedicines used in this study not only increased the effectiveness of the drugs but also further reduced the side effects. In this study, free TPEN (4.8 mg/kg and 12 mg/kg) causes hemolysis, myocardial, and liver damages, but NP@TPEN (0.6 mg/kg) treated animals have no apparent these damages and are comparative to untreated animals, which are even better than free TPEN (0.6 mg/kg) treated animals. NP@TPEN also effectively accumulated in tumor sites and removed labile Zn^2+^ induced by DOX. Thus low dose of TPEN means that the concentration of TPEN has no severe side effects *in vivo*, and the inhibitory effect on normal cells and tumor cells is very weak. But this concentration of TPEN is efficient to chelate the drug-induced labile zinc and increase the killing effect of chemotherapeutic drugs. For other tumors or clinical applications, this dose might need to be specifically optimized. In this study, we chose a ROS-responsive nanoparticle. However, ROS levels might be low in certain cancer cells, such as cancer stem cells.^33^ In this case, alternative nanoparticles (such as acid-responsive) might be included to deliver TPEN into the tumor. DOX-induced labile Zn^2+^ in tumor cells allows lung cancer cells to evade the cytotoxic effects of chemotherapeutic drugs. It has been reported that zinc plays multifaceted roles in tumors.^34^ Zn^2+^ transport via ZIP6 and ZIP10 enhances the motility of MCF-7 cells.^35^^,^^36^ In our previous study, labile Zn^2+^ assists lung cancer cells in resisting the cytotoxic effects of DOX.^19^ However, it is unknown how chemotherapy affects zinc dyshomeostasis in tumor. Many studies have reported the increase of intracellular labile Zn^2+^ in cells under stress. The changes in intracellular pH leading to the release of labile Zn^2+^ from cytosolic zinc-cysteine complexes.^37^ Phorbol 12-myristate 13-diacetate (PMA) can induce the increase of labile Zn^2+^ in fibroblasts and monocytes.^38^^,^^39^ In this study, we find that chemotherapeutic drug DOX and PTX induce the increase of intracellular labile Zn^2+^ in lung cancer cells. It is known that ROS can induce increase of intracellular labile Zn^2+^, and DOX stimulation has been linked to the excessive accumulation of ROS.^11^ Therefore, it is possible that DOX-induced ROS may be responsible for the increase of intracellular labile Zn^2+^. Our results also demonstrate that DOX-induced labile Zn^2+^ increases ABCB1 to help tumor cell to evade the cytotoxic effects of DOX.

ABCB1 is key mediator of zinc-mediated protection against to drug cytotoxicity in tumor cells. ABCB1, as an ATP-binding cassette (ABC) transporter, exhibits overexpression in various cancers, leading to resistance against chemotherapeutic drugs with diverse structures.^40^^,^^41^ Specifically, the elevated levels of ABCB1 compromise the efficacy of drugs, such as DOX, EGFR tyrosine kinase inhibitors, and cisplatin in lung cancer.^19^^,^^42^^,^^43^ Although activated by different factors, phosphorylation of AKT regulates ABCB1 transcription, while AKT inhibitors downregulate ABCB1 expression and prevents the occurrence of drug resistance.^44^^,^^45^^,^^46^ As a member of the ATP-binding cassette transporter family, ABCB1’s function is closely related to ATP. ATP can bind to ABCB1, providing energy for its pumping of chemotherapy drugs.^47^ ABCB1 has also been associated with cell stemness and is considered one of the indicators of cell stemness.^48^ Both as a tumor cell resistance and stem cell indicator, ABCB1 is positively correlated with poor prognosis.^49^^,^^50^ Our results indicate that the drugs induce the increase of labile Zn^2+^ in lung cancer cells, leading to an increase in AKT phosphorylation to upregulate ABCB1 at protein expression level, and to ATP production that enables ABCB1 to obtain more energy at the metabolic level. However, ATP, as one of the substances necessary for cell survival, it might also be involved in other processes of chemotherapy resistance, which merits further study.

NP@TPEN is potentially used in combination with chemotherapeutic drugs to overcome clinical chemoresistance of lung cancer. A meta-analysis finds that serum copper/zinc ratio is significant higher in patients with advanced stage of lung cancer than that in patients with early stage of lung cancer.^51^ It has been reported that SLC39A10 promotes the metastasis of breast cancer and the malignant progression of gastric cancer by regulating the inflow of zinc.^52^ ABCB1 is highly expressed in various types of cancer and serves as one of the indicators of chemotherapy resistance.^40^^,^^53^^,^^54^ Therefore, clinical translation study that using NP@TPEN at low dose to treat resistant tumor merits further study. TPEN can enhance the therapeutic effect of DOX in both nude mice and conventional mice, which suggests that T cells are not necessary for the enhancement effect of TPEN on chemotherapy. It is well-known that immune activation is important for long-term tumor chemotherapy.^55^ We speculate that the absorption of nanoparticle-encapsulated TPEN by T cells might be minimal, and a large number of antigens would be exposed after tumor killing by drugs, thus possibly activating the anti-tumor immune response of T cells.

In conclusion, our study provides a strategy to enhance the killing effect of DOX on lung cancer cells by chelating labile Zn^2+^ using nanosized TPEN at low dose, and avoids severe side effects. This may inspire a new approach for the clinical treatment of lung cancer with chemotherapy resistance.

### Limitations of the study

In this study, we demonstrate that two ABCB1 substrates, DOX and PTX, induce the increase of labile Zn^2+^ in tumor cells. It is still unknown whether other ABCB1 substrates could induce similar effects. Furthermore, it needs further study that whether NP@TPEN could be broadly used in ABCB1-related chemoresistance. In addition, how chemotherapeutic drugs cause an increase in labile Zn^2+^ in cells and where these Zn^2+^ are released from are questions that need to be further explored.

## Resource availability

### Lead contact

Further information and requests for resources should be directed to and will be fulfilled by the lead contact, Zhihai Qin (zhihai@ibp.ac.cn.).

### Materials availability

This study did not generate unique reagents.

### Data and code availability

• All data reported in this paper will be shared by the lead contact upon request.
• No new code was generated in this study.
• Any additional information required to reanalyze the data reported in this paper is available from the lead contact upon request.

## Acknowledgments

We thank Ningjing Lei, Linyu Zhu, Anqi Li, Xinran Zhu, and Zhenzhen Pan for their helpful discussions. We would like to thank Editage (www.editage.cn) for English language editing.

Funding: 10.13039/501100001809National Natural Science Foundation of China [grant number 32370973, 82073231 to Q.Z., 82372911 to N.C.]. Key Project of Medical Science and Technology of Henan Province [grant number SBGJ202302037, YQRC2023005, 232102311050 to N.C.].

## Author contributions

Conceptualization: Z.Q., C.N., Y.L., and L.W.; methodology: L.W., C.N., Y.L., K.Z., X.L., Y. Yan, and K.L.; investigation: L.W., C.N., Y. Yang, and Y.D.; visualization: L.W., R.C., J.W., X.Y., and X.D.; supervision: Z.Q.; writing—original draft: L.W. and C.N.; writing—review and editing: Z.Q., C.N., L.W., Y.L., and F.W.

## Declaration of interests

The authors declare no competing interests.

## STAR★Methods

### Key resources table

**Table:** 

REAGENT or RESOURCE	SOURCE	IDENTIFIER
**Antibodies**		

Phospho-histone H2A.X(Ser139)(20E3)Rabbit mAb	CST	Cat# 9718; RRID: AB_2118009
Anti-Ki67 antibody	Abcam	Cat# ab16667; RRID: AB_302459
AKT(pan)(40D4)Mouse mAb	CST	Cat# 2920; RRID: AB_1147620
Phospho-AKT(Ser473)(D9E)XP Rabbit mAb	CST	Cat# 4060; RRID: AB_2315049
P-Glycoprotein Monoclonal Antibody (C219)	Invitrogen	Cat# MA1-26528; RRID: AB_795165
β-actin	ABclonal	Cat# AC038; RRID: AB_2863784

**Chemicals, peptides, and recombinant proteins**		

DOX	Sangon Biotech	A603456-0025
PTX	Sangon Biotech	P875571
TPEN	Merck	P4413
FluoZin™-3	Thermo Fisher	F24195
Cell Counting Kit-8	Dojindo	CK04
BSA	Solarbio	A8020
PBS	Hyclone	AH29787894
DMSO	Sigma-Aldrich	D2650
DMEM	Gibco	8123645
RPMI-1640	Gibco	AJ30734507
FBS	PAN	ST30-3302
Penicillin/Streptomycin	Gibco	SV30010
DMF	Aladdin-e	D112004
Triton X-100	Sigma-Aldrich	T8787
DAPI	Solarbio	C0065
Special medium for A549/DDP cells	Procell	CM-0519
RIPA lysis buffer	Solarbio	R0020

**Critical commercial assays**		

Annexin-V-FITC/PI Apoptosis Detection Kit	Vazyme	A211-01
ATP Assay Kit	Beyotime	S0026
BCA Protein assay kit	ThermoFisher	23227

**Experimental models: Cell lines**		

LLC	ATCC	CRL-1642
A549	Procell	CL-0016
H1299	ATCC	CRL-5803
A549/DDP	Procell	CL-0519

**Experimental models: Organisms/strains**		

C57BL/6 female mouse	SPF Biotechnology	N/A
BALB/c nude female mouse	SPF Biotechnology	N/A
NOD-SCID female mouse	SPF Biotechnology	N/A

**Software and algorithms**		

GraphPad Prism	GraphPad	https://www.graphpad.com/
FlowJo	BD Biosciences	https://www.flowjo.com/
Vectra 3.0	Perkin Elmer	http://www.perkinelmer.com.cn/
ImageJ	Open source	N/A

### Experimental model and study participant details

#### Cell culture

LLC, A549, and LLC/DOX cells were cultured in DMEM. H1299 cells were cultured in RPMI-1640 medium, whereas A549/DDP cell lines were cultured in specialized culture medium (Procell, CM-0519). All media were supplemented with 10% FBS and 1‰ penicillin/streptomycin. The cells were cultured in a constant temperature incubator set at 37°C, with a carbon dioxide concentration of 5%.

LLC/DOX is a cell line that is less sensitive to chemotherapy than LLC cells. Initially, 3 × 10^5^ LLC cells were seeded on a 6-well plate. After overnight growth, 0.3 μM DOX was added. Following a 24-h incubation, the medium containing DOX was removed, cells were washed with PBS, and fresh culture medium without chemotherapeutics was added. This process was repeated four times, resulting in the establishment of LLC/DOX cells with increased resistance to DOX.

#### Animals and treatments

Female C57BL/6, nude, and NOD-SCID mice aged 6–8 weeks were procured from SPF Biotechnology Co., Ltd. (Beijing, China). All mice were housed at 21°C and 50% humidity, on average, on a 12 h light/dark cycle in a specific pathogen-free facility at Zhengzhou University. Mice were randomly distributed into different groups and fed with standard chow diet. Tumor cells were injected subcutaneously into the mice, and tumor growth was monitored every 2 days. Tumor volumes were calculated as length × width^2^ × 0.5. DOX (5 mg/kg) or TPEN (0.6 mg/kg) were administered via the tail vein four times with a 2-day interval. The Ethics Committee of Scientific Research and Clinical Trial at the First Affiliated Hospital of Zhengzhou University approved this study (2021-KY-0626-002).

### Method details

#### Preparation and characterization of ROS-responsive nanoparticles

ROS-responsive nanoparticles were prepared using a typical nanoprecipitation method. The synthesized polymer PEG-PPBEM (5 mg/mL in DMF, 50 μL) was added into water (950 μL). After stirring for 2 h, the DMF was removed through dialysis (MWCO = 3.5 kDa) against DI water. The structure of the ROS-responsive nanoparticle micelles was characterized using dynamic laser scattering (DLS, Zetasizer Nano-ZS, Malvern) and transmission electron microscopy (TEM, FEI Tecnai F20, acceleration voltage = 200 kV). And the drugs (DOX and TPEN) were loaded using a similar method.^58^

#### DOX release

The release of DOX was studied using a dialysis method in PBS with 1 mM H_2_O_2_. Drug-loaded micelles were dissolved in 1 mL of the release medium, placed in the dialysis bag, and then dialyzed against 25 mL of the release medium. At determined time intervals, 5 mL of the release medium was harvested and replenished with an equal volume of fresh medium. The amount of DOX released was determined by spectrofluorimetry (λex = 480 nm, λem = 590 nm).

#### Cell viability

Briefly, 1 × 10^4^ cells were cultured overnight in 96-well plates. After 24 h of drug stimulation, cell viability was determined using a CCK-8 (Cell Counting Kit-8) detection kit (Dojindo, CK04).

#### Cellular immunofluorescence

Initially, 5 × 10^5^ tumor cells were cultured in confocal small dishes. After overnight incubation, drugs were introduced to stimulate the cells for 24 h. Following this, the medium was aspirated, and the cells were washed thrice with PBS solution. The fixed sections were treated with a 4% PFA solution for 10 min at room temperature, followed by three times PBS washes. The cell membrane was permeabilized using 1‰ Triton X-100 for 15 min, and subsequently blocked with 5% bovine serum albumin (BSA) for 30 min. Primary antibody was added and incubated overnight at 4°C. The next day, the samples were briefly rewarmed for 2 min and washed three times with PBS. Subsequently, a fluorescent secondary antibody, prepared with 5% BSA, was applied and allowed to react at room temperature for 1 h. Then, three times PBS washes were conducted. DAPI-containing dye was incubated at room temperature for 10 min, and the expression levels of γ-H2AX (1:200, 9718S, CST) and Ki67 (1:200, ab16667, Abcam) were assessed using confocal microscopy.

#### Flow cytometry

Cells (1 × 10^5^) were spread onto a 12-well plate and incubated overnight, followed by a 24-h exposure to drugs. Upon drug removal, cells underwent three times PBS washes and were subsequently collected. Fixation was performed at room temperature using 4% PFA for 15 min, followed by centrifugation. The supernatant was discarded, and the sediment was washed once with PBS. Cell membrane permeabilization was achieved with 1‰ Triton X-100 for 15 min. Subsequently, γ-H2AX (1:200, 9718S, CST) antibody or Anti-Ki67 antibody (1:200, ab16667, Abcam) was added and left to incubate overnight at 4°C. On the following day, the precipitated cells were collected via centrifugation, washed once with PBS, and then incubated with a fluorescent secondary antibody at room temperature for 1 h. After washing with PBS, the cells were resuspended, precipitated with PBS, and analyzed through flow cytometry.

#### Apoptosis analysis

Cells (1 × 10^5^) were seeded on a 12-well plate and incubated overnight, followed by a 24-h exposure to drugs. Cell apoptosis was assessed using the Annexin-V-FITC/PI Apoptosis Detection Kit (A211-01, Vazyme, China).

#### Zinc staining

Following an overnight culture, cells were subjected to medication stimulation for a specified duration. After removal, cells underwent three times PBS washes, and subsequently, FluoZin-3 (Invitrogen, F24195) prepared with serum-free DMEM, was applied. The working solution of FluoZin-3, at a concentration of 2 μM, was kept away from light at room temperature for 1 h. Following staining, cells were cleaned three times with PBS before conducting subsequent testing.

#### Zinc transfer

Tumor cells (8 × 10^3^) were individually seeded in the wells of 96-well black plates and cultured overnight. After loading with 2 μM FluoZin-3 for 1 h, the culture medium was replaced with HBSS (Hank’s Balanced Salt Solution) supplemented with 1.26 mM CaCl_2_. Subsequently, the FluoZin-3 fluorescence was promptly detected using SpectraMAX i3X (Molecular Devices, CA, USA) at 525 nm to evaluate intracellular labile Zn^2+^. Sixty seconds after addition, FluoZin-3 fluorescence readings were taken every 30 s for the specified duration to monitor changes in intracellular labile Zn^2+^ in the tumor cells. In the analysis, values were normalized by subtracting those of the blank group to evaluate intracellular Zn^2+^ levels. Cells not stained with FluoZin-3 served as F0, while FluoZin-3-stained cells were represented as F. The alteration in intracellular Zn^2+^ was calculated as ΔF/F0, where ΔF = F−F0.

#### Western blot analysis

Cell precipitation was analyzed using RIPA lysis buffer (Solarbio, R0020) to collect the supernatant. Subsequently, 10% SDS-PAGE and NC membranes were used for gel electrophoresis. The bands were then incubated with primary antibody at 4°C overnight. The following day, the bands underwent incubation with secondary antibodies at room temperature for 1 h. The following primary antibodies were used: Phospho-histone H2A.X(Ser139)(20E3)Rabbit mAb (1:800, 9718S, CST), AKT(pan)(40D4)Mouse mAb (1:1,000, 2920S, CST), Phospho-AKT(Ser473)(D9E)XP Rabbit mAb (1:1,000, 4060S, CST), ABCB1 (1:800, MA1-26528, Invitrogen), and β-actin (1:3000, AC038, ABclonal).

#### Immunofluorescence

Mouse tumor tissues were embedded in O.C.T. and frozen prior to slicing using a frozen slicer. The resultant tissue slices underwent five washes with PBS to eliminate excess O.C.T. Subsequently, the slices were fixed at room temperature with 4% paraformaldehyde for 15 min, followed by three times PBS washes, and then sealed at room temperature with 5% BSA for 30 min. The tissue was further sealed with a primary antibody at 4°C overnight. On the subsequent day, a fluorescent antibody was applied and left to incubate at room temperature for 1 h. Following staining with DAPI, the specimen was sealed and photographed using VECTRA.

#### ATP assay

Tumor cells (2 × 10^5^) were seeded on a 6-well plate overnight, stimulated with drugs for 24 h, and detected using the ATP Assay Kit (S0026, Beyotime, China).

#### Mitochondrial respiration

Approximately 2 × 10^5^ LLC cells were incubated overnight in a 6-well plate and treated with drugs for 24 h. After digestion and counting (at 2 × 10^5^ cells per well), they were transferred to XF96 Cell Culture Microplates (Agilent), and the medium was switched to XF Base Medium (Agilent). Blockers were sequentially injected through the ports of Seahorse Flux Pak cartridges. The Seahorse XF96 extracellular flux analyzer (Agilent) was utilized to measure the oxygen consumption rate.

#### Nanodrug distribution

Initially, tumor cells were subcutaneously injected into C57BL/6 mice. Subsequent to tumor growth, both free and NP groups were injected into the mice via tail veins. Live imaging of the small animals was conducted at 2, 4, 6, 8, 12, and 24 h. Following 24 h of filming, we obtained photographs of the mouse heart, liver, spleen, lungs, kidneys, and tumor tissue to assess the residual presence of nanomedicine in different organs.

#### Tumor Zn^2+^ determination via FACS

The tumor tissue was removed from the subcutaneous tissue of the mice, ground, and cut into small pieces. The tissue cells were digested using enzymes and placed on a shaking table at 37°C for 2 h, with cells being agitated every 30 min. Following digestion into a single-cell suspension, the precipitate was filtered. Subsequently, flow cytometry was conducted, either directly or after staining with FluoZin-3.

### Quantification and statistical analysis

All data were analyzed using GraphPad Prism 8.0.1 and presented as the Standard Error of Mean (SEM) for a minimum of three independent experiments. All of the statistical details of experiments can be found in the figure legends and results. T-tests were employed for the comparison of two groups, and curve comparisons were conducted using two-way ANOVA. Statistical significance was considered as *p* < 0.05. ∗*p* < 0.05; ∗∗*p* < 0.01; ∗∗∗*p* < 0.001; ∗∗∗∗*p* < 0.0001; ns, not significant.
