# Supplementary material for: Chelating drug-induced labile Zn2+ with nanoparticle-encapsulated TPEN at low dose enhances lung cancer chemotherapy through inhibiting ABCB1
Source: iScience. 2024 Oct 5;27(11):111072. doi: 10.1016/j.isci.2024.111072 (PMC11539588; doi:10.1016/j.isci.2024.111072)
Supplement: Document S1. Figures S1–S5 [file mmc1.pdf]

iScience, Volume *27*

Supplemental information

Chelating drug-induced labile Zn^{2+} with nanoparticle-encapsulated TPEN at low dose enhances lung cancer chemotherapy through inhibiting ABCB1

Linlin Wang, Chen Ni, Kaili Zhang, Yuanyuan Yang, Ruoyang Chen, Xiaohan Lou, Yan Yan, Kexin Li, Ya Dong, Xiaohan Yao, Jiajia Wan, Xixi Duan, Fazhan Wang, YongJuan Li, and Zhihai Qin

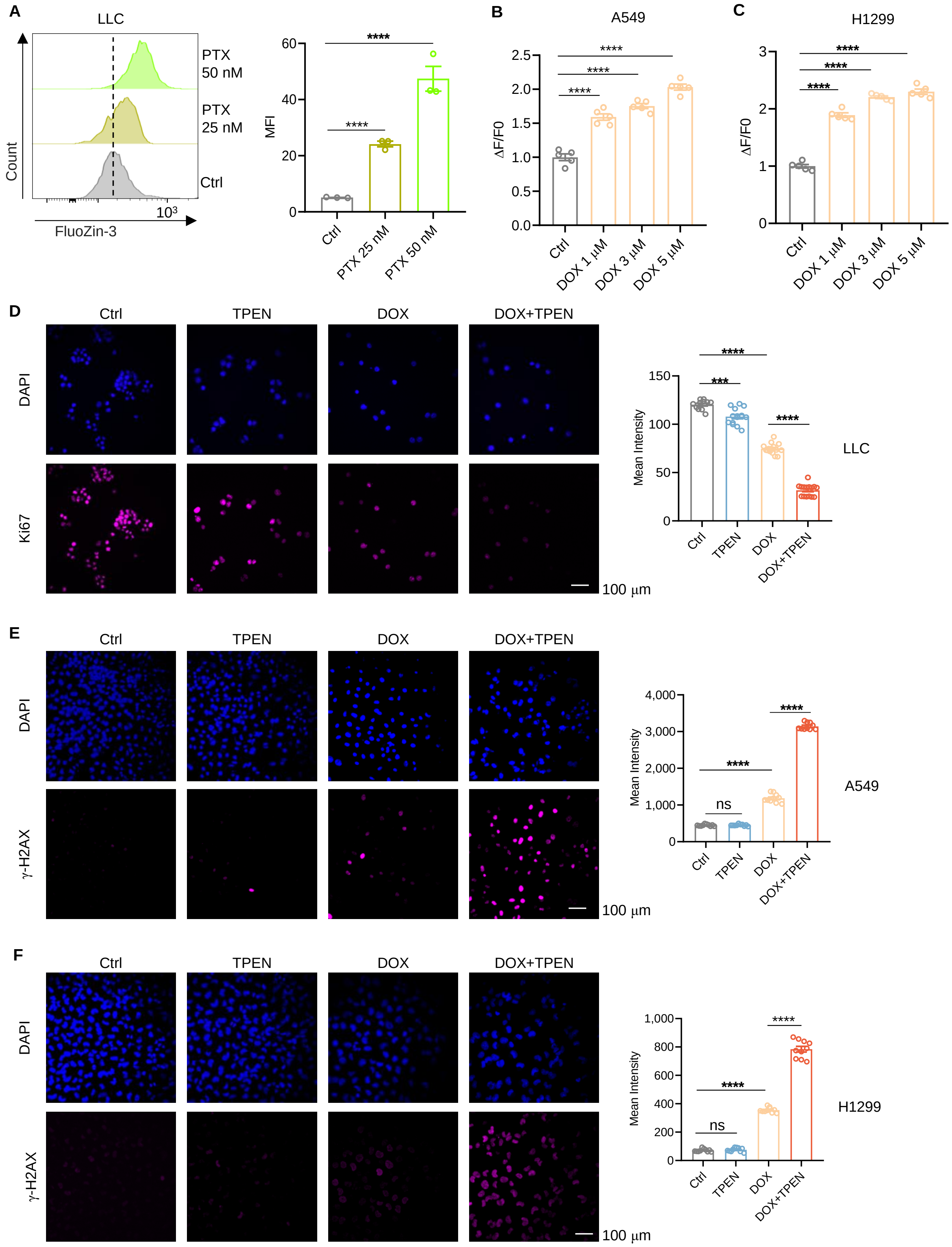

Figure S1. Paclitaxel (PTX) induced the increase of labile Zn^{2+} and TPEN enhances the proliferation inhibition and cytotoxicity of DOX in lung cancer cells. **(A)** LLC cells treated with PTX for 24 h, followed

by FluoZin-3 staining to assess intracellular zinc content (n = 3). (**B** and **C**) The levels of active zinc were labeled using FluoZin-3 staining and assessed in A549 and H1299 cells (n = 5). (**D**) CLSM images of Ki67 expression in LLC cells after treatment with 1 μM DOX and 5 μM TPEN for 24 h (n = 11 pictures per group). (**E-F**) CLSM images of γ-H2AX expression in A549 and H1299 cells after treatment with 1 μM DOX and 5 μM TPEN for 24 h (n = 10 pictures per group). Data represents the mean ± SEM of at least three replicates per condition. T-test was employed for statistical analysis. Scale bar is 100 μM. *$P < 0.05$; **$P < 0.01$; ***$P < 0.001$; ****$P < 0.0001$; ns, not significant.

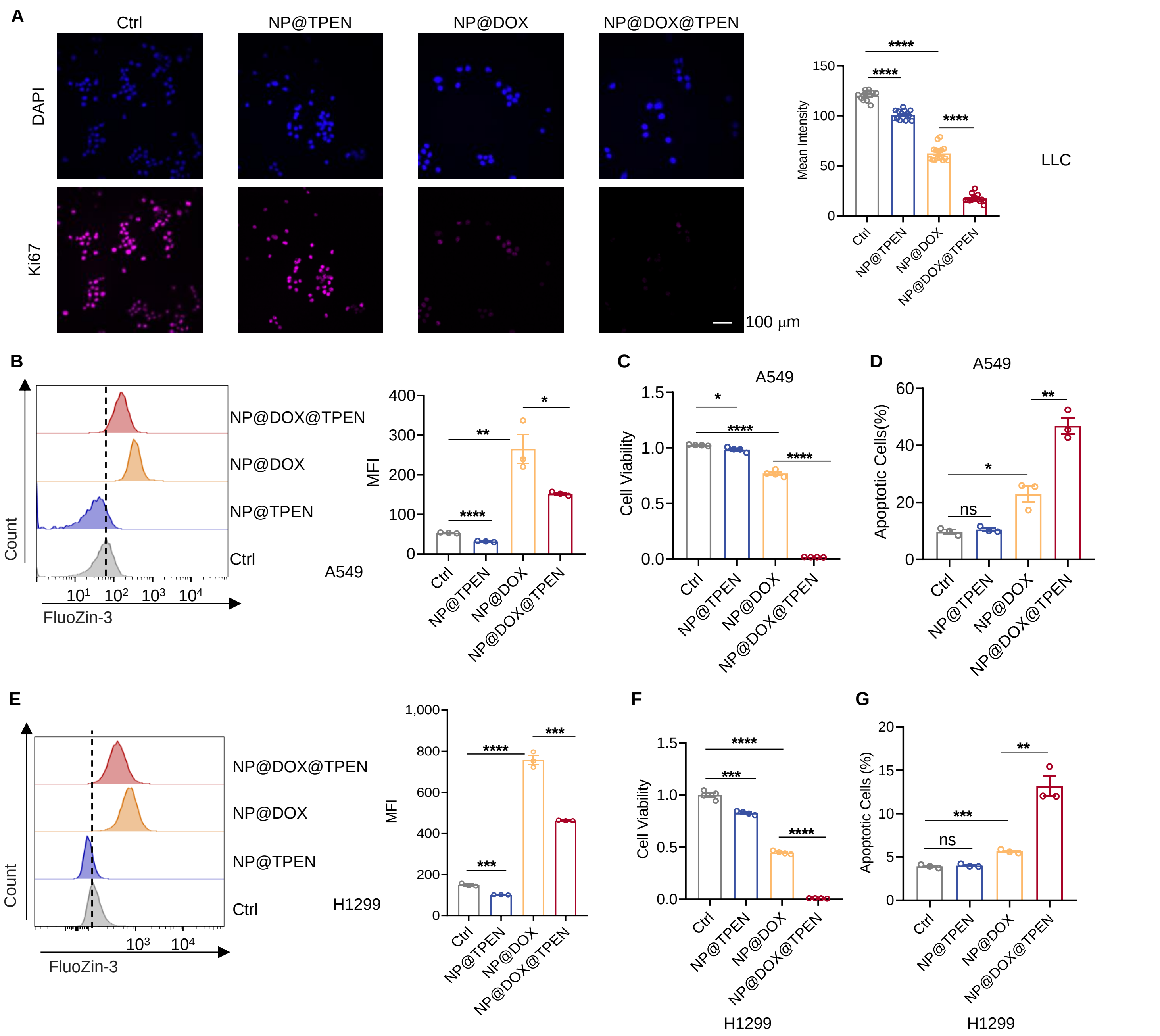

Figure S2. NP@TPEN at low dose enhanced the cytotoxicity of NP@DOX in tumor cells. (**A**) CLSM images of Ki67 expression in LLC cells after treatment with 5 μM NP@TPEN and 1 μM NP@DOX for 24 h (n = 11 pictures per group, Scale bar is 100μM). (**B and E**) Treatment with 5 μM NP@TPEN and 1 μM NP@DOX to A549 and H1299 cells for 24 h, followed by FluoZin-3 staining and flow cytometry detection of intracellular zinc content. (**C and F**) CCK-8 for detecting cell viability after treatment with 5 μM NP@TPEN and 1 μM NP@DOX in A549 and H1299 cells for 24 h. (**D** and **G**) Cell apoptosis by Flow cytometric analyses in A549 and H1299 cells, after treatment with 5 μM NP@TPEN and 1 μM NP@DOX for 24 h. Data are presented as the mean ± SEM from a minimum of three replicates per condition. B, D, E and

G: n = 3; C and F: n = 4. T-test was utilized for statistical analysis. **P* < 0.05; ***P* < 0.01; ****P* < 0.001; *****P* < 0.0001; ns, not significant.

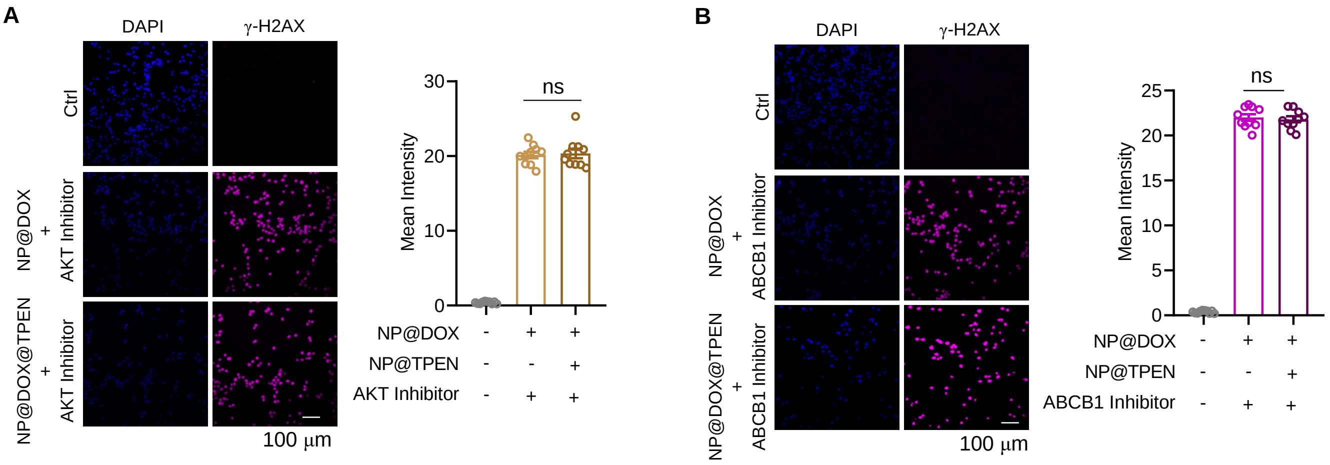

Figure S3. NP@TPEN is ineffective to enhance the cytotoxicity of NP@DOX when AKT-ABCB1 axis was inhibited in LLC cells. (**A**) CLSM images of γ-H2AX expression after treatment with 1 μM NP@DOX, 5 μM NP@TPEN, and 25μM AKT inhibitor (LY294002) for 24 h. (**B**) CLSM images of γ-H2AX expression after treatment with 1 μM NP@DOX, 5 μM NP@TPEN, 0.1μM ABCB1 inhibitor (Tariquidar) for 24 h. Data are presented as the mean ± SEM from a minimum of three replicates per condition. A and B: n = 10 pictures per group. T-test was utilized for statistical analysis. Scale bar is 100 μM. **P* < 0.05; ***P* < 0.01; ****P* < 0.001; *****P* < 0.0001; ns, not significant.

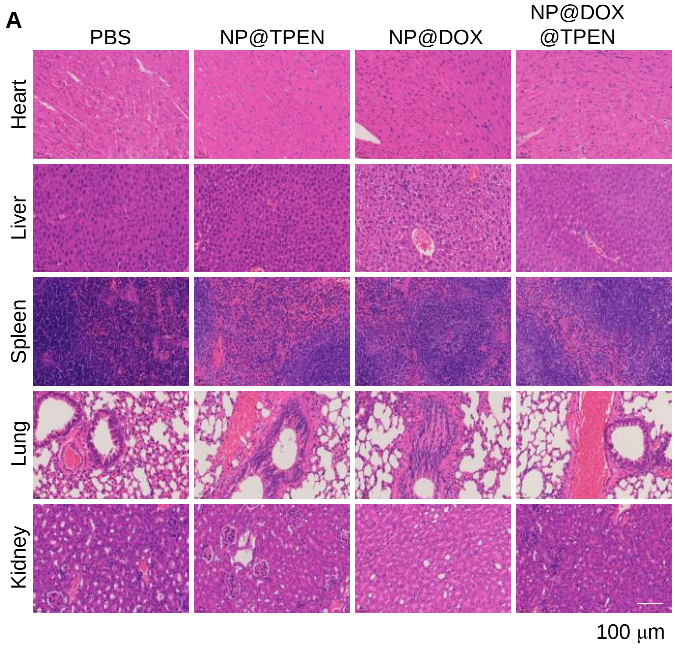

Figure S4. Organs damage after nanomedicine treatment. (**A**) HE images of mice hearts, livers, spleens, lungs, and kidneys. Scale bar is 100 μM.

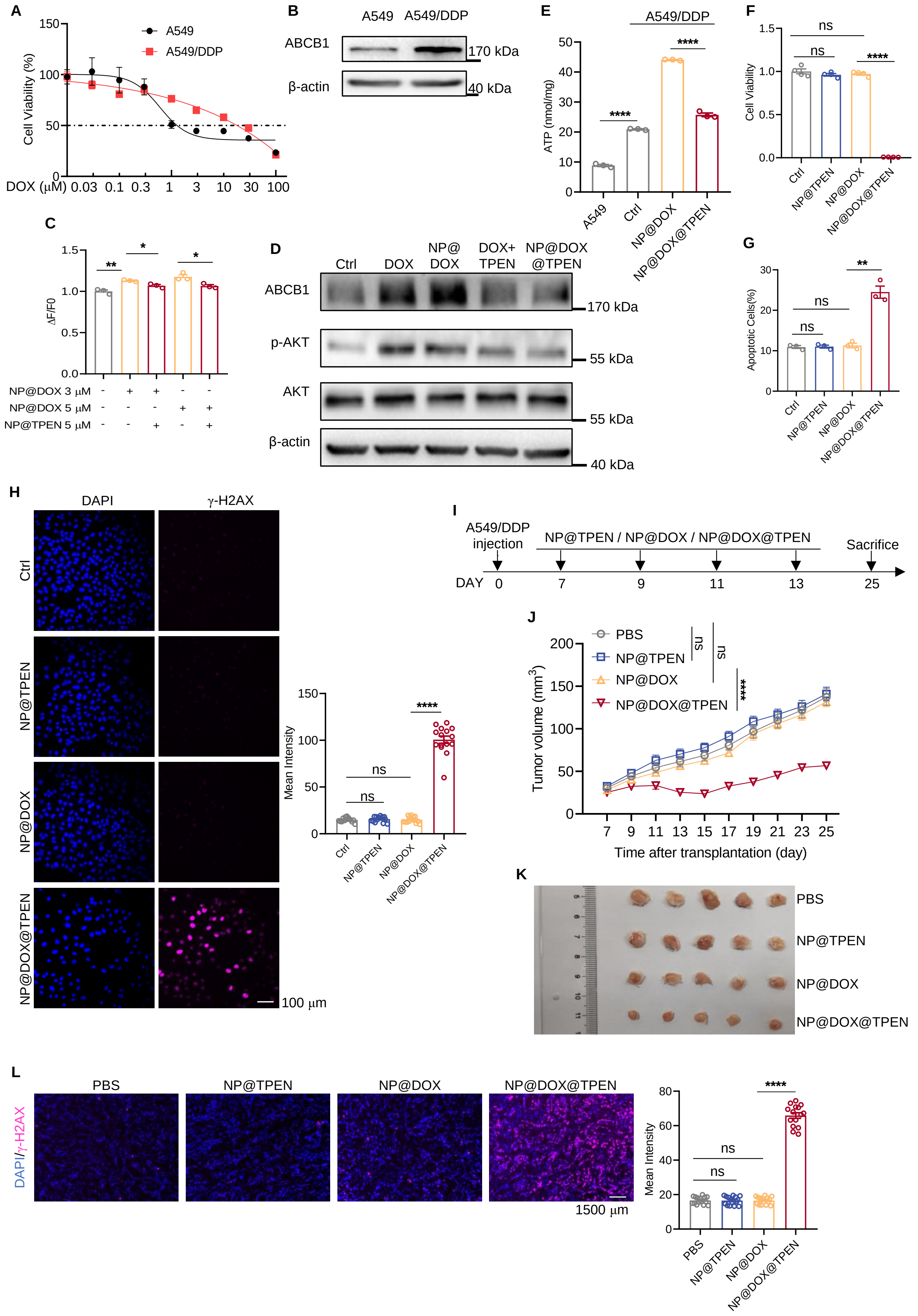
A
Cell Viability (%)
A549
A549/DDP
DOX (μM) 0.03 0.1 0.3 1 3 10 30 100
B
A549 A549/DDP
ABCB1
β-actin
170 kDa
40 kDa
C
ΔF/F0
NP@DOX 3 μM
NP@DOX 5 μM
NP@TPEN 5 μM
D
Ctrl DOX NP@DOX DOX+TPEN NP@DOX@TPEN
ABCB1
p-AKT
AKT
β-actin
170 kDa
55 kDa
55 kDa
40 kDa
E
A549/DDP
ATP (nmol/mg)
A549
Ctrl
NP@DOX
NP@DOX@TPEN
F
Cell Viability
ns
Ctrl
NP@TPEN
NP@DOX
NP@DOX@TPEN
G
Apoptotic Cells(%)
ns
Ctrl
NP@TPEN
NP@DOX
NP@DOX@TPEN
H
DAPI
γ-H2AX
Ctrl
NP@TPEN
NP@DOX
NP@DOX@TPEN
100 μm
Mean Intensity
ns
I
A549/DDP injection
NP@TPEN / NP@DOX / NP@DOX@TPEN
Sacrifice
DAY 0 7 9 11 13 25
J
PBS
NP@TPEN
NP@DOX
NP@DOX@TPEN
Tumor volume (mm3)
Time after transplantation (day)
K
PBS
NP@TPEN
NP@DOX
NP@DOX@TPEN
L
PBS
NP@TPEN
NP@DOX
NP@DOX@TPEN
DAPI/γ-H2AX
1500 μm
Mean Intensity
ns

Figure S5. NP@TPEN sensitizes resistant A549/DDP cells to chemotherapy. (**A**) The sensitivity of A549 and A549/DDP to different concentrations of DOX for 24 h. (**B**)Western blot analysis of ABCB1 expression. (**C**) The zinc inside the cells were stained with FluoZin-3 and then detected in A549/DDP cells for 24 h. (**D**) Western blot analysis of ABCB1, p-AKT, and AKT expression in A549/DDP cells. (**E**) Measurement of ATP levels in A549 and A549/DDP cells after adding 1 μM NP@DOX, and NP@DOX@TPEN (1 μM NP@DOX combined with 5 μM NP@TPEN) for 24 h. (**F**) CCK-8 for detecting cell viability after treatment for 24 h. (**G**) Cell apoptosis by flow cytometric analyses in A549/DDP cells. (**H**) CLSM images of γ-H2AX expression (Scale bar is 100 μM). (**I**) Time schedule of treatments in NOD-SCID mice. (**J-K**) Tumor volume within the 25-day observation period (n = 5 mice per group). (**L**) Immunofluorescence images of tumor sections stained with γ-H2AX (Scale bar is 1500 μM). The data represent the mean ± SEM from at least three replicates per condition. A, C, E, G: n = 3; F: n = 4; H, L: n = 15; J: n = 10 mice per group. J was analyzed by two-way ANOVA tests, and others were analyzed by T-tests. *P < 0.05; **P < 0.01; ***P < 0.001; ****P < 0.0001; ns, not significant.
